# P-44. Immunogenicity and Safety of High-dose versus Standard-dose Quadrivalent Inactivated Influenza Vaccine in Patients Living with HIV: A Randomized Controlled Trial

**DOI:** 10.1093/ofid/ofae631.251

**Published:** 2025-01-29

**Authors:** Nutchaya Laoharattanahirun, Sasisopin Kiertiburanakul, Kobporn Boonnak, Jackrapong Bruminhent

**Affiliations:** Faculty of Medicine Ramathibodi Hospital, Mahidol University, Bangkok, Krung Thep, Thailand; Faculty of Medicine Ramathibodi Hospital, Bangkok, Krung Thep, Thailand; Faculty of Medicine Siriraj Hospital, Mahidol University, Bangkok, Krung Thep, Thailand; Faculty of Medicine Ramathibodi Hospital, Mahidol University, Bangkok, Krung Thep, Thailand

## Abstract

**Background:**

Influenza can lead to severe complications in patients living with HIV (PLWH). Annual standard-dose (SD) quadrivalent inactivated influenza vaccine (QIIV) is recommended for PLWH, although it is associated with poor vaccine response. We aimed to investigate the immunogenicity and safety of high-dose (HD) versus SD QIIV in PLWH, as well as safety.

**Methods:**

We conducted a randomized, open-label, controlled trial to compare the immunogenicity of the 2023 southern hemisphere HD QIIV at 60 μg (Efluelda, Sanofi) versus SD QIIV at 15 μg (Fluarix tetra, Sanofi) in patients living with HIV (PLWH) during August and November 2023. Seroprotection (SP), defined as serum hemagglutination-inhibition antibody titers ≥1:40 at 1 month post-vaccination, was assessed. Adverse events (AEs) following vaccination were also evaluated.

**Results:**

We enrolled 110 patients who received the study vaccine, and 103 (52 HD; 51 SD) were eligible for analysis. Among them, 65 (62.1%) were male, with a mean age (SD) of 49.6 (11.2) years and a median CD4 count (IQR) of 555 (418-680) cells/mm^3^. The integrase strand transfer-based regimen was the most common antiretroviral regimen (57.3%), and the majority had an undetectable HIV viral load (93.2%). Fifty-two (50.5%) achieved SP to all strains (A/H1N1, A/H3N2, B/Victoria and B/Yamagata) after vaccination. Those who received HD QIIV reached SP significantly more compared to SD QIIV (61.5% vs. 39.2%, p = 0.02). In particular, SP to the A/H3N2 strain was significantly greater in the HD QIIV group compared to the SD QIIV group (76.9% vs. 52.9%, p = 0.011). Use of HD QIIV were independent factors for SP to all strains (OR 2.7, 95%CI 1.9-6.1, p = 0.02) and SP to the A/H3N2 strain (OR 3.2, 95%CI 1.4-7.8, p = 0.01). Twenty-nine (57.3%) participants in the HD group reported AEs, including muscle ache (36.5%), pain at the injection site (21.2%), and fever (13.5%). Those were all non-severe and comparable to those receiving SD QIIV.

**Conclusion:**

HD QIIV could offer significantly better immunogenicity across all strains, especially the A(H3N2) strain, compared to SD QIIV in PLWH and should be considered as the preferred influenza vaccine for this population, without safety concerns. TCTR20230501002.

**Disclosures:**

**All Authors**: No reported disclosures

